# Comparison between linear mixed model and threshold model in the estimation of variance components in age at first calving and milk production in buffaloes

**DOI:** 10.3389/fvets.2025.1649690

**Published:** 2025-09-02

**Authors:** Raimundo Nonato Colares Camargo-Júnior, Cláudio Vieira de Araújo, Marina de Nadai Bonin Gomes, José Ribamar Felipe Marques, Welligton Conceição da Silva, Carlos Eduardo Lima Sousa, Rubens Lima de Andrade, Albiane Sousa de Oliveira, Éder Bruno Rebelo da Silva, Jaqueline Rodrigues Ferreira Cara, José de Brito Lourenço-Júnior, Alison Miranda Santos, André Guimaraes Maciel e Silva

**Affiliations:** ^1^Postgraduate Program in Animal Science (PPGCAN), Institute of Veterinary Medicine, Federal University of Para (UFPA), Federal Rural University of the Amazon (UFRA), Brazilian Agricultural Research Corporation (EMBRAPA), Castanhal, Brazil; ^2^Federal Institute of Education, Science and Technology of Pará (IFPA), Santarém, Brazil; ^3^Department of Agricultural and Environmental Sciences, Federal University of Mato Grosso (UFMT), Sinop, Brazil; ^4^Meat Technology and Processing Laboratory of the College of Veterinary Medicine and Animal Science, Universidade Federal do Mato Grosso do Sul, Campo Grande, Brazil; ^5^Embrapa Eastern Amazon, Belém, Brazil; ^6^Department of Veterinary Medicine, University Center of the Amazon (UNAMA), Santarem, Brazil

**Keywords:** Bayesian inference, Geweke test, Markov chains via Monte Carlo, Murrah breed, Gibbs sampler

## Abstract

The genetic evaluation of Murrah buffaloes can be optimized by associating milk production, genetic value of sires, and age at first calving. Therefore, the aim of this study was to compare the Linear Mixed Model with the Threshold Model and their genetic association with milk production and the genetic evaluation of sires in the estimation of variance components of age at first calving in Murrah buffalo. The dataset comprised information on total milk production and age at first calving of Murrah buffaloes. The mixed linear animal model, designated as Model 1, was employed to estimate variance components. In a subsequent analysis, designated as Model 2, the age at first calving was examined in conjunction with the milk production. The variance components were obtained by Bayesian inference, using the Gibbs sampler to obtain posterior means. The *t*-test was then applied in order to compare the means of two samples. The additive genetic correlations between milk production and age at first calving were low in both models, with values equal to 0.11 and 0.17 for Models 1 and 2, respectively. The descriptive analysis of the predicted breeding values revealed that, irrespective of the model, the values for milk production exhibited minimal variation. In a separate analysis, Model 2 exhibited a reduced amplitude for age at first calving and enhanced prediction accuracy, particularly for sires with negative breeding values for this trait. Consequently, the Threshold Model strategy for analyzing age at first calving variance components is more efficient than a Linear Mixed Model. It provides more accurate genetic value estimates for sires without affecting milk production predictions.

## 1 Introduction

Research on Murrah buffaloes is justified by their importance as a dairy breed, entailing physiological and management complexities inherent to dairy buffalo farming. The relentless pursuit of high milk yields in Murrah buffaloes often is associated with specific reproductive challenges, such as prolonged anestrus, silent estrus, and reduced conception rates. These challenges directly affect overall productivity by prolonging calving intervals and decreasing lifetime production. Consequently, it is essential to focus jointly on reproductive disorders, along with the nutritional and management demands specific to the productive and reproductive needs of Murrah buffaloes. This joint approach can significantly increase reproductive efficiency and overall productivity in this specific breed. This, in turn, has the potential to generate substantial benefits for this segment of the buffalo agribusiness.

It is imperative to underscore certain limitations, including the occurrence of calving in buffaloes. As in cattle, challenges often arise, primarily dystocia due to fetal-maternal disproportion and nutritional deficiencies that lead to conditions such as placental retention or puerperal paresis (1, 2). These complications result in increased intervals between calving, compromised subsequent fertility, and high calf mortality (3, 4). Effective peripartum nutrition management and meticulous prepartum assessment are crucial (5, 6). Such difficulties can have a significant impact on herd productivity and economic viability, necessitating proactive clinical management (7).

Strategic supplementation with specific feed additives critically enhances buffalo reproductive health (8). Essential trace minerals (e.g., Se, Cu, Zn, Mn), vital vitamins (e.g., A, E, D), and omega-3 fatty acids optimize ovarian function and conception rates (5, 9). These nutrients bolster immune response, mitigate postpartum disorders, and improve overall reproductive efficiency (6, 10). By correcting nutritional deficiencies, these additives support the high metabolic demands of reproduction, making their precise dietary integration paramount for sustainable breeding success (4, 6).

During late gestation, particularly the final trimester, buffaloes are susceptible to prevalent metabolic diseases, including ketosis, hypocalcemia, and acidosis (4, 11). These conditions arise from metabolic imbalances like energy deficits, calcium dysregulation, or altered ruminal pH (8, 11, 12). Such disorders severely compromise maternal health, impair immune function, and jeopardize fetal viability. Consequently, parturition success, subsequent lactation, and reproductive performance are critically impacted, necessitating proactive nutritional and management interventions to mitigate adverse outcomes (13).

These interventions can and should benefit from promising research results in dairy farming. For example, it has been demonstrated in dairy calf breeds (14) that organic mineral supplementation is an effective strategy for improving mineral bioavailability and supporting the health of these animals during their first years of life. In another analysis, a feed additive mixture of condensed and hydrolysable tannins (15) improved reproductive efficiency in lactating cows, reducing the number of services per conception.

Other favorable results include the use of new methodologies, such as mid-infrared spectroscopy (16), to predict blood metabolic indicators in Holstein cow milk, which has proven to be an alternative for identifying cows at risk of negative energy balance and subclinical ketosis. Similarly, feed additives with immunomodulatory capabilities (17) are also emerging as strategies to improve metabolic and immunological responses to subacute ruminal acidosis in dairy cows.

Beyond these immediate clinical and nutritional interventions, the long-term genetic progress of a herd critically relies on the rigorous genetic evaluation of breeding animals, such as buffalo bulls (18, 19). It allows the selection of the best animals, resulting in superior productivity and quality (20).

By analyzing performance data and, with increasing frequency, genomic information, the genetic value of breeding animals can be estimated, reflecting their capacity to enhance characteristics such as milk production and age at first calving (21–23).

Consequently, selection for increased milk production is predicted to have a positive effect on the age at first calving, as demonstrated in Nili-Ravi (24) and Murrah (25) buffaloes. In other analyses, Tamboli et al. (26), Seno et al. (25), Mathur and RoyChoudhury (27), and Calanni Macchio et al. (28) evaluated data from Murrah buffaloes and Italian buffaloes. These analyses demonstrated that the opposite is also true; that is, selection by age at first calving showed improvement in milk production and other characteristics. This was evidenced by a negative association between age at first calving and standard milk production in the first lactation, productive life, total milk production throughout life, standard milk production throughout life, and reproductive efficiency. The findings of these studies indicate that decreasing the age at first calving would enhance various performance traits throughout the animal’s lifespan, extending beyond merely milk production.

It is imperative to note that this genetic evaluation is predicated on the precise quantification of genetic and environmental influences. Consequently, the estimation of variance components is essential. This approach enables precise measurement of additive genetic variance, as well as other crucial factors such as environmental variance and genotype-Environment interaction, as emphasized by Ranjan et al. (29) and Zhang et al. (30).

In addition to variance components, environmental factors, including calving period and season (31, 32) and age at first calving (32), were identified as significant determinants of performance. Furthermore, advanced evaluations consider direct, maternal, and permanent maternal effects (33) and employ methods such as Principal Component Analysis (33) to assess genetic trends and relationships between genetic values.

Among the widely used methods for estimating these components, the Linear Mixed Model and Threshold Models stand out. These are statistical tools used to analyze dependent data, i.e., data where observations are related or influence one another (34, 35). The aim was to compare the Linear Mixed Model with the Threshold Model and their genetic association with milk production and the genetic evaluation of sires in the estimation of variance components of age at first calving in Murrah buffalo.

## 2 Materials and methods

The analysis was based on a dataset comprising 2,866 records of total milk production and 661 records of age at first calving for 1,122 Murrah buffaloes participating in the Brazilian Buffalo Improvement Program (PROMEBULL).

Total milk production (TMP) was regressed as a function of lactation length (LL). Subsequently, the TMP was corrected for 305 days of lactation using the expression: MP305 = TMP + (5.42705) (305 − LL). This expression, referred to as “total milk yield,” offers a succinct representation of the relationship between TMP and LL. Consequently, MP305 is regarded as the rectified value for total milk yield, which will be utilized for subsequent analysis. This value is here in after referred to as milk production (MP).

Direct heterozygosity effect (HTZ) was estimated using breed information, defined as the deviation from the Murrah breed, which varies from zero (0) to one (1) (36).

The heterozygosity value (HTZ) was calculated using the equation (37) as follows:


HTZ=(MS×OM)+(MM×OS)

Where:

MS is the degree of racial composition of the Murrah breed from the sire’s contribution.

OM is the degree of racial composition of the other breed from the dam’s contribution.

MM is the degree of racial composition of the Murrah breed from the dam’s contribution.

OS is the degree of racial composition of the other breed from the sire’s contribution.

The calving months were grouped into four calving seasons (CS) to adapt the analysis to regional climatic adversities, considered as follows: CS = 1 the period from January to March; CS = 2 the period from April to June; CS = 3 the period from July to September; and CS = 4 the period from October to December.

Contemporary groups were a combination of the fixed effects of herd, year, and CS. Contemporary group information with at least three observations was included.

The mixed linear animal model, designated as Model 1, was employed to estimate variance components. Subsequently, the method was employed to forecast breeding values, which are denoted as:


y=Xβ+Za+e

Where:

𝑦 is a vector of observations.

𝛽 is a vector of fixed effects (group of contemporaries and linear effect of the heterosis covariate).

𝑎 is the vector of direct additive genetic effect.

𝑒 is the vector of residual effect.

𝑋 is the incidence matrix that associates 𝛽 with 𝑦.

𝑍 is the incidence matrix of the direct genetic effect.

The age at first calving (AFC) was subsequently categorized into two classes. Class 1 encompassed values below the median, defined as less than or equal to 36.32 months of age. Class 2 comprised values greater than or equal to the median. In a subsequent analysis, designated as Model 2, the AFC was examined in conjunction with the MP. The AFC was conceptualized as a dichotomous variable, while the MP was considered a continuous variable within a Threshold Model. It was hypothesized that the scale underlying the AFC exhibited a continuous normal distribution and was represented as:


$$U|\theta \prime \sim \mathrm{NW},I\sigma_{e}^{2}$$

Where:

U is the base scale vector; θ′ is the vector of location parameters (the true ‘unobserved’ breeding values); W is the incidence matrix; I is the identity matrix, $\sigma_{e}^{2}$ is the residual variance.

The (co)variance components were obtained by Bayesian inference, using the Gibbs sampler with the GIBBS1F90 program (38) to obtain posterior means. A chain size of 600,000 cycles was used, with cycles saved every 20 cycles and a discard period of 60,000 initial cycles. The Geweke criterion (39) was employed to assess the quality of the chains, with a 5% probability level set as the cutoff.

In order to compare the posterior heritability means between the models, 30 sub-samples of 2,700 cycles were randomly obtained from the Gibbs chains for each model. The Gibbs chains were used to generate the posterior heritability means. The heritability averages were obtained from these subsamples, resulting in 30 values for each model. The *t*-test was then applied to compare the means of two samples, with a significance level of 0.05.

Subsequent to acquiring the predicted breeding values for MP and AFC for the sires, the Spearman correlations were calculated in three distinct scenarios and for each model. In the initial scenario, the sample was comprised of all sires with progenies exhibiting production. In the second scenario, the consideration was limited to sires with positive progenies for MP. Finally, the third scenario encompassed sires with progeny that were negative for AFC.

The estimated means and standard deviations of the minimum and maximum values observed for MP and AFC are shown in Table 1.

**Table 1:** Number of observations (*N*), estimates of means and standard deviation (SD), minimum (Min) and maximum (Max) values for MP and AFC.

Trait	*N*	Mean	SD	Min	Max
MP	2,866	2281.97	570.47	868.20	4162.80
AFC	661	37.39	5.42	25.00	67.00

MP = milk production and AFC = age at first calving.

## 3 Results and discussion

The estimation of the additive and residual genetic variance components for milk production (MP) and age at first calving (AFC) was demonstrated to be statistically robust using Bayesian inference with Markov chains via Monte Carlo (MCMC). The convergence of the chains was assessed by the Geweke test, whose values were found to be close to zero and with associated *p*-values exceeding 0.05 for all parameters. This indicates satisfactory convergence at the 5% significance level (see Table 2 for details).

**Table 2:** The posterior means, along with their respective SD, are presented for the variance components of additive genetic and residual effects.

	Mean	SD	CI (95%)		Geweke
Model 1					
Additive genetic variance					
MP	817.4838	99.24689	619.6	1,005	0.03
AFC	7.3456	2.755	2.251	12.59	−0.02
Residual variance					
MP	1694.51	58.7792	1813.0	2363.8	−0.02
AFC	19.4667	2.4177	14.8	24.24	0.02
Model 2					
Additive genetic variance					
MP	820.33	99.697	624.93	1015.70	0.01
AFC	0.6845	0.4948	−0.2852	1.6543	−0.04
Residual variance					
MP	1694.5	58.957	1578.9	1810	−0.01
AFC	1.0077	0.0364	0.9363	1.079	0.01

The component credibility interval (CI) and Geweke’s test, along with the associated probability for MP and AFC, are also included.

In Model 1, an additive genetic variance of 817.48 was observed for MP and 7.35 for AFC, with residual variances of 1694.51 and 19.47, respectively. In a separate analysis, Model 2 revealed that MP maintained comparable estimates for genetic (820.33) and residual (1694.5) variance, while AFC exhibited a substantial reduction in the genetic (0.68) and residual (1.01) components.

As demonstrated in Table 3, the posterior means for heritabilities are consistent across models for milk production, with a mean of 0.32 ± 0.03. However, for AFC, the estimates exhibited greater variability. The mean values obtained for Models 1 and 2 were 0.27 ± 0.09 and 0.37 ± 0.13, respectively. A *t*-test performed with independent subsamples of the Gibbs chain demonstrated a statistically significant discrepancy between the models for AFC (*t* = 187.53; *p* < 0.0001).

**Table 3:** The posterior means of heritability, with respective standard deviations, are presented on the diagonal for Model 1 and Model 2 (in parentheses), with the posterior means of additive genetic correlation for the Model 1 (above the diagonal) and Model 2 (below the diagonal) for milk production (MP) and age at first calving (AFC).

Trait	MP	AFC
MP	0.32 ± 0.03 (0.32 ± 0.03)	0.11 (0.17)
AFC	0.19 (0.18)	0.27 ± 0.09 (0.37 ± 0.13)

MP = milk production and AFC = age at first calving.

The additive genetic correlations between MP and AFC were low in both models, with values equal to 0.11 and 0.17 for Models 1 and 2, respectively. Spearman correlations between the estimated breeding values (see Table 4) provide further evidence for this pattern, with values ranging from 0.24 (Model 1) to 0.38 (Model 2). There is also high consistency between models in the ordering of the sires within each trait (*ρ* = 0.99 for MP and 0.72 for AFC).

**Table 4:** Estimates of Spearman’s correlations between the breeding values of sires for MP and AFC are presented for Models 1 and 2, respectively, with the correlations between models illustrated on the diagonal and those between models on the above and below the diagonal.

Trait	Trait	
	MP	AFC
MP	0.99	0.24
AFC	0.38	0.72

MP = milk production and AFC = age at first calving.

The descriptive analysis presented in Table 5 revealed the descriptive analysis of the predicted breeding values revealed that, irrespective of the model, the values for MP demonstrated minimal variation. In a separate analysis, Model 2 exhibited a reduced amplitude for AFC and enhanced prediction accuracy, particularly for sires with negative breeding values for this trait. In all situations analyzed, the accuracy for MP remained constant (0.70–0.72), in contrast to the increase observed for AFC, which rose in Model 2 from 0.49 to 0.55.

**Table 5:** Descriptive analysis of the Number of observations (N), minimum (Min) and maximum (Max) predicted breeding values of sires for MP and AFC are presented for Models 1 and 2, respectively.

Position	Trait	N	Breeding values					Accuracy
			Mean	SD	Min.	Max.	SD/Mean	
Model 1								
1	MP	81	−3.1260	19.4845	−48.0932	45.6233	−6.2330	0.72
	AFC	81	−0.0382	1.4435	−2.6844	5.9047	−37.7880	0.49
2	MP	33	15.3209	11.8880	0.2522	45.6233	0.7759	0.72
	AFC	33	0.1875	1.5855	−2.4968	5.9047	8.4560	0.47
3	MP	44	−8.1503	19.3613	−48.0932	33.7785	−2.3755	0.70
	AFC	44	−1.0384	0.7131	−2.6844	−0.0698	−0.6867	0.48
Model 2								
1	MP	81	−3.2645	19.6075	−48.2807	45.9139	−6.0063	0.72
	AFC	81	−0.0129	0.2172	−0.4491	0.7339	−16.8372	0.55
2	MP	33	15.1039	12.1405	−48.2807	45.9139	0.8038	0.72
	AFC	33	0.0611	0.2160	−0.4491	0.7339	3.5352	0.54
3	MP	42	−9.3460	16.8426	−48.2800	16.7867	−1.8021	0.70
	AFC	42	−0.1680	0.1157	−0.4490	−0.0077	−0.6887	0.54

The results demonstrated the robustness of the Bayesian approach in the inference of genetic components, as observed in other surveys (40, 41), particularly with regard to MP in cattle and buffaloes (42–45). In other words, the direct comparison made in this study between Linear Mixed Model and Threshold Models for the age at first calving in Murrah buffaloes, highlighting the Threshold Model as the best for evaluating the sires, represents a significant methodological contribution.

This assertion aligns with the conclusions of Colonia et al. (43), who previously corroborated the efficacy of Threshold Models in the analysis of non-normally distributed characteristics in dairy cattle, notwithstanding divergent prior specifications. While Camargo Júnior et al. (42), Kumar et al. (44), and Mendes-Malhado et al. (45) also employed Bayesian inference to estimate genetic parameters in dairy cattle (milk and growth traits), their focus differs, as they did not make this specific comparison between Linear Mixed Model and Threshold Models for discrete traits.

The low genetic correlations (0.11–0.17) observed between milk production and age at first calving in this study offer specific insights into Murrah reproduction. These insights are contextualized by the broader utility of Bayesian methodologies highlighted by Sun et al. (40) and van de Schoot et al. (41) for complex genetic analyses. The stability observed in the estimates of additive genetic variance and heritability between the two models evaluated suggests that this trait has a robust and well-defined genetic basis.

This consistency, even with different residual structures and parameterizations, indicates that MP is less susceptible to variations in the statistical model. This finding provides substantial credibility to genetic predictions for milk production (MP) and underscores its viability as a selection criterion in genetic enhancement programs for dairy buffalo farming (30, 46–48).

By contrast, AFC was highly sensitive to model specification. There was a marked reduction in additive genetic variance in Model 2, and the heritability estimates differed significantly. This discrepancy can be attributed to the trait’s lower heritability, its intrinsic biological complexity, and the greater influence of environmental and non-genetic factors (20, 26, 49, 50).

The greater accuracy of the Threshold Model for AFC possibly derives from its inherent ability to more effectively capture the underlying distribution of the characteristic when treated categorically, even though it is a biologically continuous trait. By dichotomizing AFC (below or above the median), the Threshold Model can effectively map crucial biological or management thresholds that a Linear Mixed Model would disregard. This approach makes the Threshold Model less sensitive to noise and nonlinearities often present in complex reproductive traits that are strongly influenced by environmental and non-genetic factors.

According to the results obtained, in the case of AFC, noise could come from several sources, such as small variations in nutritional management between animals, seasonal climatic differences not fully captured, data recording errors, or even small individual biological fluctuations that do not have a significant genetic component. By treating AFC as a categorical variable (e.g., early or late using the median as the threshold), the Threshold Model focuses on the classification that is most robust to random disturbances, filtering out the noise and highlighting the most important signal of precocity. Consequently, the model refines the signal-to-noise ratio, focusing on the qualitative outcome of early versus late calving, which has great practical relevance in buffalo selection.

Despite the similarity in the ratio between the standard deviation and the mean of heritability across models, indicating relative consistency in variability, the magnitude of the differences in means underscores the substantial impact of model alterations on genetic estimates for AFC. The present findings highlight the imperative for judiciousness in statistical modeling of reproductive traits, which characteristically manifest elevated environmental variability and diminished direct genetic control (44, 51, 52).

Additionally, it was evident that Model 2 showed a substantial reduction in genetic and residual variances for AFC. This suggests that the Threshold Model was more efficient in discriminating the true sources of variation, resulting in more accurate estimates of additive genetic effects and, consequently, greater predictive accuracy. This is because the Threshold Model deals with non-linearities by assuming that there is a continuous latent variable (not directly observable) – the predisposition or liability to AFC, which follows a normal distribution but manifests itself in observable categories when a certain threshold is exceeded.

By dichotomizing AFC, the Threshold Model implicitly recognizes that genetic impact can be better understood in terms of crossing a threshold. It can therefore better capture the idea that each additional day in AFC can change significantly once certain critical points (the thresholds) are reached, offering a more realistic representation of the biological complexity of this trait. The threshold model design assumes a continuous underlying predisposition or liability, but observes a categorical outcome, proving more suitable for addressing the biological complexity and low heritability typical of reproductive traits.

The estimates of additive genetic and Spearman rank-order correlations between MP and AFC were low. This finding indicates that there is a weak genetic association between these two traits. In practice, this indicates that ranking sires based on genetic merit for one trait does not serve as a reliable predictor of performance for the other (53, 54). Therefore, it is imperative to adopt selection indices that consider both traits jointly and in balance, to avoid limited or even undesirable gains in non-target traits (55–57).

The almost perfect consistency in the ranking of sires for MP between models (*ρ* = 0.99) confirms the robustness of genetic inference for this trait. This finding indicates that the selection of superior animals remains consistent, even when the model is modified. In another analysis for AFC, although the Spearman correlation between models was moderately high (*ρ* = 0.72), greater sensitivity to the adopted specifications was evident, indicating that minor changes to the model can alter the genetic ranking of individuals. This is particularly salient in the context of reproductive traits, where variations in the residual structure or the incorporation of supplementary effects can markedly enhance the precision of estimates (58–60).

Another salient point pertains to the analysis of the accuracy of genetic predictions. While the accuracy of MP remained stable for both models and the different sire subsets evaluated, Model 2 demonstrated significant enhancement in AFC accuracy. This finding indicates that the careful selection of the appropriate residual structure can facilitate the extraction of more valuable information from the database and improve the reliability of estimates for traits that are difficult to measure or have low heritability. Additionally, the diminished range of breeding values observed in Model 2 may suggest a more conservative yet precise modeling approach, a strategy that is particularly advantageous in selection contexts where there is a risk of overestimating genetic merit.

Consequently, the necessity of meticulous statistical modeling tailored to the biological characteristics of the studied traits is evident. While MP is a highly stable trait from a genetic point of view, AFC requires more refined analytical strategies that may include environmental, genomic, or management information to better capture its complexity (19, 61). In this sense, the utilization of Bayesian inference in conjunction with convergence verification and the implementation of statistical tests on Gibbs chain samples has been demonstrated to be a potent and adaptable approach (62–66). This approach has been demonstrated to produce reliable point estimates, thereby facilitating more informed decisions in the context of selection programs.

## 4 Conclusion

Overall, the Threshold Model strategy demonstrated superior efficiency in extracting variance components for AFC when directly compared to its treatment as a continuous variable within a Linear Mixed Model. The Threshold Model provided more accurate genetic value estimates for sires without affecting MP predictions.

The heritability estimates for MP indicate the possibility of substantial genetic progress through selective breeding. However, sires that demonstrated favorable genetic merit for precocity do not necessarily exhibit the greatest merit for MP.

In the pursuit of furthering the modeling of AFC, prospective investigations could entail the incorporation of more intricate environmental data, including temperature-humidity indices, precipitation, and variations in pasture quality or nutritional management during pivotal phases of animal development. This approach would facilitate the capture of environmental changes that exert a significant influence on AFC.

At the genomic level, the application of high-density SNP arrays or whole-genome sequencing would enable the identification of specific genes or Quantitative Trait Loci (QTLs) associated with precocity in buffaloes. The integration of these genomic data with advanced statistical methodologies, such as single-step genomic best linear unbiased prediction (ssGBLUP), holds considerable promise in enhancing the precision of genetic value predictions for AFC in buffalo. This approach would serve to reinforce and complement the advancements already demonstrated by the threshold model.

## Data Availability

The datasets presented in this study can be found in online repositories. The names of the repository/repositories and accession number(s) can be found in the article/supplementary material.
